# Biological and Physical Environmental Drivers of Diet Variation in Northern Fur Seals

**DOI:** 10.1002/ece3.71998

**Published:** 2025-08-18

**Authors:** Elizabeth A. McHuron, Jeremy T. Sterling, Katie Luxa, James Thorson, Rod Towell, Rolf R. Ream, Tonya Zeppelin

**Affiliations:** ^1^ Cooperative Institute for Climate, Ocean, and Ecosystem Studies University of Washington Seattle Washington USA; ^2^ Marine Mammal Laboratory, Alaska Fisheries Science Center, National Marine Fisheries Service, National Oceanic and Atmospheric Administration Seattle Washington USA; ^3^ Resource Ecology and Fisheries Management, Alaska Fisheries Science Center, National Marine Fisheries Service, National Oceanic and Atmospheric Administration Seattle Washington USA

**Keywords:** Bering Sea, *Callorhinus ursinus*, frequency of occurrence, laaqudan, Pribilof Islands

## Abstract

The eastern Bering is a productive high‐latitude ecosystem characterized by high interannual variability in physical environmental conditions that impact biological communities. We investigated how the diet composition of northern fur seals ( *Callorhinus ursinus* ) breeding on the Pribilof Islands was influenced by this variation, focusing on water temperatures (surface and bottom) and an index of walleye pollock abundance within foraging areas. We also explored whether interannual variation in diet composition influenced fur seal pup mortality rates or body mass. The frequency of occurrence (FO) of all eight fur seal prey groups detected from hard parts analysis of samples collected from 1987 to 2012 was affected by interannual variation in at least one of the three environmental variables. Pollock was the predominant prey group across the study years, highlighting the importance of this species to Pribilof Island fur seals. Not only was pollock consumed more frequently as it became more abundant within fur seal foraging areas, but its relative abundance also affected how frequently other prey groups were consumed. A considerable amount of variation in FO of almost all prey groups was explained by year effects, suggesting that water temperatures alone were not sufficiently capturing the influences of regional and local physical environmental conditions on prey availability for fur seals. The summed FO of non‐pollock prey groups had a small but detectable effect on the mass of male pups, indicating that the availability of prey groups beyond just pollock is somewhat beneficial for female northern fur seals early in lactation. Our results suggest that projected environmental changes in the eastern Bering Sea are likely to influence fur seal diets, but predicting the magnitude and direction of such changes is hampered until the underlying drivers of the observed temporal trends are better resolved.

## Graphical abstract

Interannual variation in northern fur seal diet composition was influenced by physical and biological variables within local foraging areas in the eastern Bering Sea. Our findings indicate that projected environmental changes in the eastern Bering Sea are likely to affect fur seal diet composition and potentially increase trophic pressure on non‐pollock prey species.

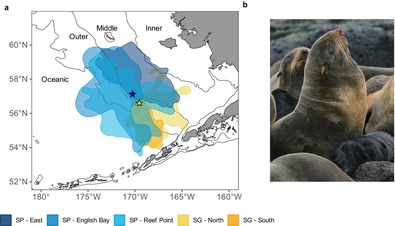

## 1 Introduction

Diet studies provide insight into a species' ecological role, overlap of shared resource use with other species, and vulnerability to natural and human‐induced fluctuations in prey resources (Azevedo et al. 2006; Gibson et al. 2018; Lacher Jr et al. 2019). Environmental changes, such as those arising from anomalous events, recurring climate patterns, and interannual variation, often impact predator diet because they alter prey abundance and distribution (Brodeur et al. 2007; Ancona et al. 2012; Thomsen et al. 2018; Kliska et al. 2022). Such diet changes can impact predator health, reproductive success, and survival through a multitude of pathways, including changes in body condition, altered exposure to predators or anthropogenic activities, and shifts in contaminant exposure (McKinney et al. 2013; Gladics et al. 2015). They can also alter the strength of top‐down forcing on prey populations, which may have broader implications for ecosystem structure and management efforts (Caut et al. 2008; Smith et al. 2021; Gómez‐Alceste and Rando 2024). Understanding the relationships between predator diet composition and environmental variables is thus important for climate‐informed species‐ and ecosystem management, particularly when prey species are of economic interest.

Northern fur seals ( *Callorhinus ursinus* ) are seasonal mammalian residents of the eastern Bering Sea, a high‐latitude ecosystem that supports a diverse biological community and valuable fishing industries (Springer et al. 1996). During the summer and fall, females adopt a central‐place foraging strategy while rearing pups, alternating nursing visits on land with foraging trips at sea. The physical environment of the eastern Bering Sea during the summer and fall is strongly influenced by winter sea ice dynamics, resulting in a pool of cold summer bottom water on the continental shelf. The southern extent of this cold pool varies, leading to “cold” and “warm” years that affect many eastern Bering Sea prey species (Wyllie‐Echeverria and Wooster 1998; Kotwicki and Lauth 2013; Duffy‐Anderson et al. 2017). Longer‐term climate patterns, such as the Pacific Decadal Oscillation and North Pacific Gyre Oscillation, and regime shifts also influence physical and biological conditions on the Bering Sea shelf (Benson and Trites 2002; Danielson et al. 2011). Like many marine predators, northern fur seals exhibit diet variation across their foraging range (Zeppelin and Orr 2010). Such variation is particularly noticeable on the Pribilof Islands, where lactating females from different breeding rookeries use distinct foraging areas of the continental shelf and deep ocean basin (Robson et al. 2004), giving rise to dietary differences at relatively fine spatial scales (Zeppelin and Ream 2006). Temporal variation in diet is also present but less well documented than spatial patterns (Antonelis et al. 1997; Zeppelin and Ream 2006; Sinclair et al. 2008).

The Bering Sea has experienced anomalous years of warm sea surface temperatures and record sea ice lows in the last decade (Stabeno and Bell 2019), with oceanographic warming projected to increase considerably in the next century (Hermann et al. 2019). These anomalous conditions strongly impacted Bering Sea biological communities (Duffy‐Anderson et al. 2019; Siddon et al. 2020) and led to rapid borealization of Arctic communities (Fossheim et al. 2015). Ecosystem projections based on underlying climate models predict considerable declines in fish biomass (Holsman et al. 2020; Whitehouse et al. 2021), including for walleye pollock ( *Gadus chalcogrammus* ; hereafter pollock). This species is the primary prey of northern fur seals foraging on the eastern Bering Sea continental shelf and the target of one of the largest U.S. commercial fisheries. Northward shifts in pollock distribution during recent warming events (O'Leary et al. 2020), coupled with projected declines in biomass, are likely to lead to reduced availability for fur seals on the Pribilof Islands, with unknown effects on fur seals or potential alternative prey species.

The objectives of this study were to (i) identify relationships between northern fur seal diet composition on the Pribilof Islands and environmental conditions within fur seal foraging areas, and (ii) determine whether diet composition influences fur seal pup body mass and mortality rates. To accomplish this, we took advantage of existing long‐term datasets on northern fur seal diet and pup metrics that spanned several decades. Fur seals are an abundant predator in the eastern Bering Sea—current population estimates are ca. 600,000 individuals (Young et al. 2023)—that exert considerable top‐down forcing on key prey populations (McHuron et al. 2020). Despite this, the fur seal population breeding in the Bering Sea (the Eastern Pacific stock) has experienced ongoing declines since the mid‐ to late‐1990s (Young et al. 2023). Initial declines were driven by losses on St. Paul and St. George Islands, two of the Pribilof Islands, but population trends on St. George stabilized in the mid‐2000s while St. Paul Island continues to decline. Results from this study are thus informative not only for understanding contemporary drivers of dietary variation, but also provide insight into how projected environmental changes may influence interactions with prey species and any potential adverse effects of projected pollock declines on the Pribilof Island fur seal population.

## 2 Methods

All work described below was conducted in accordance with the Marine Mammal Protection Act under National Marine Fisheries Service (NMFS) Permits # 598, 837, 782‐1455, and 782‐1708. An Institutional Animal Care and Use Committee was established for the Alaska Fisheries Science Center in 2010; all experimental protocols used in this study were reviewed and approved by the committee.

### 2.1 Sample Collection and Processing

**FIGURE 1:**
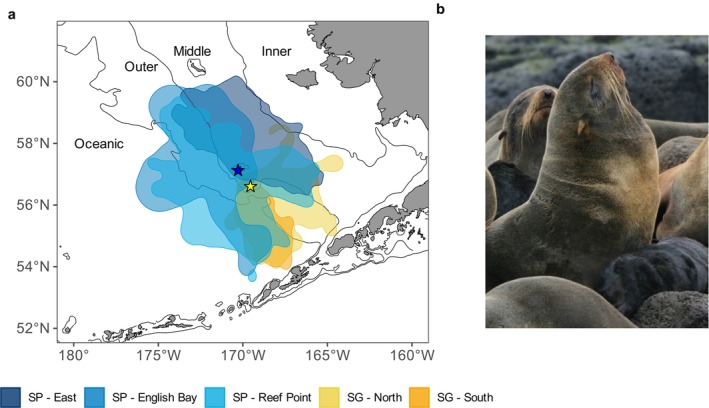
(a) August foraging areas of lactating northern fur seals at each rookery complex on St. Paul Island (blue star, SP) and St. George Island (yellow star, SG). Foraging areas are based on 95% utilization distributions derived from satellite telemetry data. Black lines (from right to left) represent the 50, 100, and 200 m isobaths that separate the inner, middle, and outer shelf, and oceanic basin. (b) Adult female northern fur seals and pups on the Pribilof Islands. Photo credit: NOAA Fisheries/Alaska Ecosystems Program.

Northern fur seal fecal (scat), regurgitation (spew), and enema samples were collected in August from 1987 to 2012 at breeding rookeries on St. Paul Island and St. George Island (Figure 1). Scat and spew samples were opportunistically collected; it is assumed that their collection proportion reflects the natural frequency of occurrence of scats and spews on rookeries. Enemas were administered to adult females captured in conjunction with other research activities by introducing ~1 L warm water into the colon via the anus and collecting the naturally expelled material (Staniland et al. 2003). The sex and age class associated with rookery scat and spew samples are unknown, although they are likely to predominantly reflect adult female diet. Juveniles and subadult males primarily use haul‐out sites that are spatially separate from breeding areas, and scat from adult males is easily recognized and likely to be minimal given their presence is reduced after early August following the commencement of breeding.

Once collected, samples were stored frozen until processing, where they were washed in nested sieves (minimum mesh size 0.5 mm). Staff at Pacific Identifications Inc. (Victoria, British Columbia) and the Marine Mammal Laboratory (Seattle, WA) used hard parts, including fish bones, fish otoliths, and cephalopod beaks, to classify prey to the lowest taxonomic level possible, which ranged from species to class (Table S1). Samples were assigned to one of five rookery complexes previously defined for the Pribilof Islands (Figure S1), which are collections of terrestrial breeding sites in close geographical proximity that share similarities in diets (Zeppelin and Ream 2006).

The diet composition at each rookery complex was described using frequency of occurrence (FO)

$$\mathrm{FO}=\frac{\sum_{k=1}^{s}I_{\mathrm{ik}}}{s}$$

where $I_{\mathrm{ik}}$ equals either 1 if at least one individual of species i is present in sample k, or 0 otherwise, and s is the total number of samples. Frequency of occurrence was calculated for each species grouping (Table S1), which for some groups consisted of a single species (e.g., pollock) but for others contained groupings of several taxonomically related species due to rarity or difficulty in identifying to the species level (e.g., salmonids). This metric of diet composition is relatively simplistic as it does not account for numerical importance or mass contribution of a given prey species. We chose to use FO instead of metrics that rely on estimates of species abundance or size because it allowed for the greatest sample sizes (i.e., not all hard parts were enumerated or measured in every sample) and the resulting estimates are mathematically independent (i.e., they are not compositional). We combined data from all sample types (scat, spew, enema) to provide a more complete representation of diet, since larger prey and cephalopods are often regurgitated (Gudmundson et al. 2000). Years with < 40 samples at a given complex were excluded from further analysis (Figure S2). Key prey groups were identified as those where the mean FO across all years was ≥ 0.05 at one or more complexes. More general prey groups, such as gadids or cephalopods, were not included despite meeting the 0.05 threshold as key prey because of low taxonomic resolution.

### 2.2 Environmental Data

We explored the influence of three physical and biological environmental variables—sea surface temperature (SST), bottom temperature, and an index of pollock abundance—on northern fur seal diet composition (also referred to more generally throughout as “environmental” variables). We focused primarily on physical environmental variables because regular targeted surveys of northern fur seal prey species were limited, and water temperatures are well documented as influential on the distribution and/or abundance of many eastern Bering Sea fish species (Kotwicki and Lauth 2013; Thorson et al. 2017; Barbeaux and Hollowed 2018; Yasumiishi et al. 2020). Bottom temperatures were obtained from the eastern Bering Sea bottom trawl survey conducted annually in June and July, available as an interpolated temperature raster from the “coldpool” package (Rohan and Barnett 2023). For SST, we used the NOAA 1/4° Optimum Interpolation Sea Surface Temperature (OISST) v2 (accessed from https://psl.noaa.gov/thredds/catalog/catalog.html), produced as a monthly mean. We restricted SST data to locations on the continental shelf for consistency with variables derived from the bottom trawl survey. The pollock abundance index was calculated from the number of pollock per area trawled (no km^−2^) during the bottom trawl survey (available from https://www.fisheries.noaa.gov/foss). Pollock abundance indices derived from surface and midwater trawls were not included here because data were not available across the entire time series of fur seal diet samples. The pollock abundance index was considered a predictor variable for all prey groups because it is the most frequently consumed species by fur seals, and we hypothesized its availability may drive consumption of other species. We spatially linked environmental datasets to complex‐specific 95% utilization distributions of lactating female northern fur seals to calculate complex‐specific means for each year. Utilization distributions were calculated with the “adehabitatHR” package (Calenge 2024) using August satellite telemetry data from 299 adult female fur seals instrumented between 1992 and 2018 (Figure 1).

### 2.3 Pup Mass and Mortality Data

Pup body mass and mortality data are two of the best long‐term datasets available for Pribilof Island fur seals to evaluate whether variation in lactating female diet composition may have implications for population dynamics. Pup masses predominately reflect the provisioning ability of the female between birth and the time of sampling. Pup mortality rates in part reflect female provisioning, predominately her success during the first foraging trip to sea (Trites 1992), and emaciation comprises approximately 53% of early pup mortality on St. Paul Island (Spraker and Lander 2010). Other causes of mortality include trauma, perinatal mortality, and infectious diseases. These two metrics are thus complementary and were not correlated in our dataset.

Pup mass data have been collected annually or biennially in mid to late August on the Pribilof Islands since the 1980s, with a few exceptions. Pup sex is also recorded at the time of sampling. Because pups are not weighed on the same day in each year, we used linear regressions to estimate daily average growth rates and standardize pup mass to the same day within each year (the median sampling day of August 25). The response variable for each regression was pup mass, and the predictor variable was day (a value from 1 to 31). Separate regressions were conducted for each complex and pup sex. The regression coefficient (the slope) was multiplied by the number of days between the measurement and August 25 and then added (or subtracted) from the measurement. Sex‐specific means and standard deviations were calculated for each year and complex, excluding pups weighed before August 19 and after August 31 to further minimize the effects of sampling date on body mass. Plots revealed that average standardized mass was anomalously high in 1994 at St. George complexes; values were ~2 kg higher than in all other years and far outside any interannual variation present at either island. These values were excluded from further analysis because of concern about the validity of these measurements.

Counts of dead pups have been conducted annually or biennially in late August at select rookeries on each island as part of routine population monitoring. Counts were conducted at multiple rookeries within each complex prior to 2006; whereas counts were conducted at one or two rookeries within each complex from 2006 onwards. Pup mortality rates were calculated as the number of dead pups relative to the estimated total number of pups born on each rookery. A complete description of methodology and sampling schedules in each year can be found in Alaska Fur Seal Investigation Reports provided by NOAA (https://www.fisheries.noaa.gov/alaska/marine‐mammal‐protection/alaska‐fur‐seal‐investigations‐decade).

### 2.4 Statistical Analyses

We used generalized additive models (GAMs) implemented in “mgcv” (Wood et al. 2016) to explore the influence of biological and physical environmental variables on diet composition (FO) for each prey group. Each predictor variable was modeled as a smooth term fitted with a thin plate spline and a binomial distribution using a logit link. Additional variables in each model included a smoothed effect of year and a random effect of complex (intercept only, bs = “re”). The sample size was included as a weighting factor since the response variable was provided as the number of occurrences/the number of samples instead of as a matrix of presence and absences. In all models, we included a penalty for non‐informative smooths (select = T), weighted based on the number of samples, and used REML for estimating smooth parameters. Model assumptions were checked via residual plots and functions within the “mgcv”, “gratia” (Simpson 2023), and “DHARMa” (Hartig 2022) packages. Assumption checking revealed the addition of year created high concurvity in the models for all predictor variables (worst > 0.9), which describes the situation when a smooth term can be approximated by some combination of others. Despite this, we retained the year term because (1) its inclusion largely had a minimal effect on smooths of other variables, (2) plots of residuals (from models without year) against this term indicated its inclusion was warranted, and (3) high concurvity estimates were typically due to the combination of multiple variables and not just a single variable.

Relationships between pup metrics (mass, mass standard deviation, mortality rates) and diet composition were also assessed using GAMs. In mass analyses, exclusion of data collected at St. George complexes in 1994 resulted in the loss of two data points (instead of four), as there was only paired diet data at SG—North in this year. To limit the number of predictor variables, we focused just on pollock and a single metric representing other prey groups, represented as the summed FO of all other prey groups. The FO of pollock, summed FO of other prey, and year were modeled as smooth terms fitted with a thin plate spline and Gaussian distribution. In mass analyses, separate smooths were fit for each sex using the factor by smooth notation. Additional variables in each model included random effects of complex and rookery (intercept only, bs = “re”), and a fixed effect of sex (mass analyses only). In all models, we included a penalty for non‐informative smooths (select = T) and used REML for estimating smooth parameters. Model assumptions were checked as described for the diet composition analysis. As in the diet composition analysis, the full models had high concurvity, but all variables were retained for similar reasons described previously.

We considered predictor variables to be important contributors in analyses when (i) the approximate significance of the smooth term was < 0.05 and (ii) 95% confidence intervals of the fitted smooth did not contain zero across the entire range. The relative importance of each predictor variable was calculated using the “gam.hp” package (Lai et al. 2024), which accounts for the unique and shared variance between predictor variables due to concurvity. All analyses were conducted using R v. 4.4 (R Core Team 2024).

## 3 Results

**FIGURE 2:**
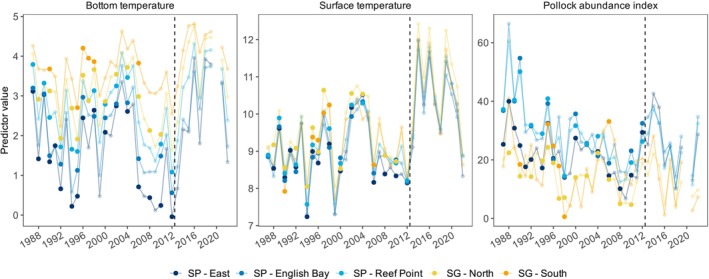
Temporal variation in bottom temperature (°C), surface temperature (°C), and the pollock abundance index (1000s km^−2^) within foraging ranges of lactating adult females from the Pribilof Islands. Points are colored by rookery complex. Transparent points indicate years not included in the model, either because of no data or because sample sizes were < 40. Values to the right of the dashed line indicate more recent years outside of the range of the dataset.

Yearly mean (±SD) sample sizes at each rookery complex were 64 ± 21 (SG—South), 97 ± 38 (SG—North), 70 ± 23 (SP—Reef Point), 103 ± 49 (SP—English Bay), and 117 ± 46 (SP—East). On average, scat samples comprised most sample types at all complexes (complex means = 78.6%–95.1%), followed by spews (3.9%–20.5%), and enemas (< 1%–1.0%). Spew samples occurred more frequently in the St. George (19.4%–20.5%) compared with St. Paul datasets (3.9%–7.7%), presumably reflecting St. George Island fur seals' greater consumption of prey items such as squid that are frequently regurgitated. Enema samples were only collected in 4 years, comprising < 1%–10% of the sample composition of a complex in those years. Key prey groups included pollock, Pacific herring ( *Clupea pallasii* ), hexagrammid/sablefish (Hexagrammidae and *Anoplopoma fimbria*), salmon (predominately *Oncorhynchus* spp.), sand lance (*Ammodytes* spp.), northern smoothtongue ( *Leuroglossus schmidti* ), Gm/Gm ( *Gonatus madokai* and *G. middendorffi* ) and Gb/Bm ( *Gonatopsis borealis* and *Berryteuthis magister* ; Table 1). There was considerable temporal and spatial variation in the values of biological and physical environmental variables during years with diet samples (Figure 2). Temporal trends were generally similar among complexes and reflected the warm and cold periods characteristic of the eastern Bering Sea continental shelf during the summer.

**TABLE 1:** Summary of total sample sizes, number of years with data, and frequency of occurrence (FO, %) for key prey groups consumed by northern fur seals from the Pribilof Islands in August, separated by rookery complex. Sample size represents the total number of samples across all years, with the minimum and maximum per year in parentheses. Frequency of occurrence values represent the average of yearly FO, with minimum and maximum values per year in parentheses.

	St. George Island		St. Paul Island		
	South	North	Reef Point	English Bay	East
Sample size	386 (40–98)	1358 (47–185)	841 (43–119)	1343 (42–194)	1984 (43–207)
Number of years	6	14	12	13	17
Key prey groups					
Walleye pollock	43.2 (25.0–61.3)	62.4 (29.3–84.3)	75.8 (57.4–96.2)	76.8 (64.7–92.8)	72.4 (52.6–91.8)
Hexagrammid/sablefish	3.3 (1.4–5.3)	4.4 (0.0–24.1)	7.3 (0.0–23.5)	7.9 (0.0–22.2)	6.6 (0.0–26.6)
Sand lance	3.8 (0.0–12.0)	3.3 (0.0–8.1)	10.6 (0.0–22.2)	7.3 (0.0–16.7)	15.1 (1.3–35.7)
Salmon	25.5 (12.5–32.5)	23.7 (6.4–39.7)	11.5 (0.0–24.6)	12.8 (1.4–37.3)	12.3 (5.6–21.7)
Pacific herring	1.5 (0.0–7.1)	4.5 (0.0–14.9)	4.1 (0.0–12.2)	5.6 (0.0–25.5)	9.5 (0.0–46.0)
Northern smoothtongue	16.6 (5.7–27.5)	5.2 (0.0–14.9)	5.6 (1.1–13.4)	2.5 (0.0–9.0)	1.4 (0.0–5.1)
Gm/Gm^a^	11.7 (1.4–27.1)	5.8 (1.1–13.3)	11.3 (0.0–28.8)	15.0 (0.0–35.6)	7.5 (0.0–23.6)
Gb/Bm^b^	47.9 (37.3–60.4)	21.7 (8.6–35.2)	12.5 (3.4–19.2)	6.7 (2.0–10.2)	3.2 (0.0–13.6)

^a^ *Gonatus madokai* / *G. middendorffi* .

^b^ *Gonatopsis borealis* / *Berryteuthis magister* .

### 3.1 Diet and Environment

**FIGURE 3:**
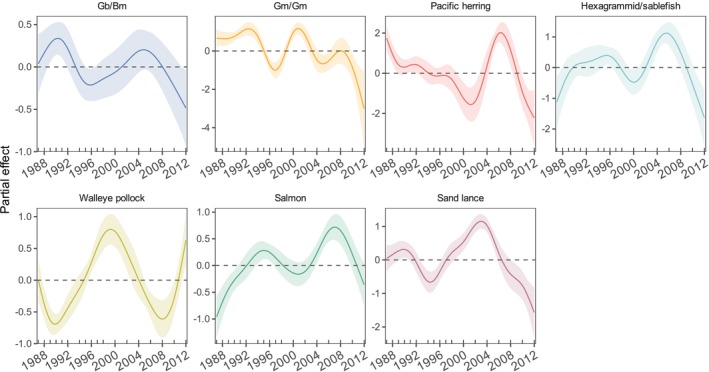
Partial effects of year on the frequency of occurrence of key prey groups in northern fur seal diets from the Pribilof Islands. The shaded area is the approximate 95% point‐wise confidence interval. Each subplot represents a different key prey group. Results are displayed on the link function scale. Prey abbreviations: Gb/Bm = *Gonatopsis borealis/Berryteuthis magister*, Gm/Gm = *Gonatus madokai/G. middendorffi*.

The deviance explained by models ranged from 70.4% to 92.8% (Table 2). A considerable amount of the explained deviance was attributed to either complex or year for almost all prey groups. Complex was a significant predictor of FO for all prey groups except herring, although its relative contribution varied among prey groups. For example, complex had a much greater contribution to the deviance explained than all other important variables for Gb/Bm (43.7%), but much lower contributions for Gm/Gm (11.3%) and the hexagrammid/sablefish group (12.5%, Table 2). Year was significant in explaining variation in FO for all prey groups except northern smoothtongue, with consistently high relative contributions to the explained deviance for all remaining prey groups (27.6%—42.3%) except Gb/Bm (7.9%). Temporal patterns of Pacific herring, salmon, sand lance, and hexagrammid/sablefish tended to be similar post‐2000, with increased FO generally coinciding with the warm phase in the early 2000s followed by decreased FO during the subsequent cold phase in the late 2000s (Figures 2 and 3).

**TABLE 2:** Summary of generalized additive model outputs for relationships between northern fur seal prey frequency of occurrence and biological and physical environmental variables (Bottom = bottom temperature, Surface = surface temperature, Pollock = pollock abundance index). The percent deviance explained by models is shown (total) alongside the percent contribution of each variable to the explained deviance, which represents the sum of the unique and shared variance between predictor variables. Bolded numbers represent variables considered to be important predictors of diet composition for a given prey group.

Key prey group	% Deviance					
	Total	Bottom	Surface	Pollock	Year	Complex
Walleye pollock	82.2	**14.1**	**10.9**	**12.6**	**35.4**	**26.9**
Hexagrammid/sablefish	82.1	**22.4**	9.9	**24.3**	**30.9**	**12.5**
Sand lance	83.6	15.4	17.1	**13.5**	**27.6**	**26.4**
Salmon	70.5	**15.2**	10.2	**23.8**	**32.2**	**18.6**
Pacific herring	85.9	**30.1**	**11.8**	**6.6**	**40.4**	11.1
Northern smoothtongue	70.4	20.6	**35.5**	5.9	7.5	**30.5**
Gm/Gm^a^	86.6	**13.0**	**19.5**	13.9	**42.3**	**11.3**
Gb/Bm^b^	92.8	27.0	**14.2**	7.2	**7.9**	**43.7**

^a^ *Gonatus madokai* / *G. middendorffi* .

^b^ *Gonatopsis borealis* / *Berryteuthis magister* .

**FIGURE 4:**
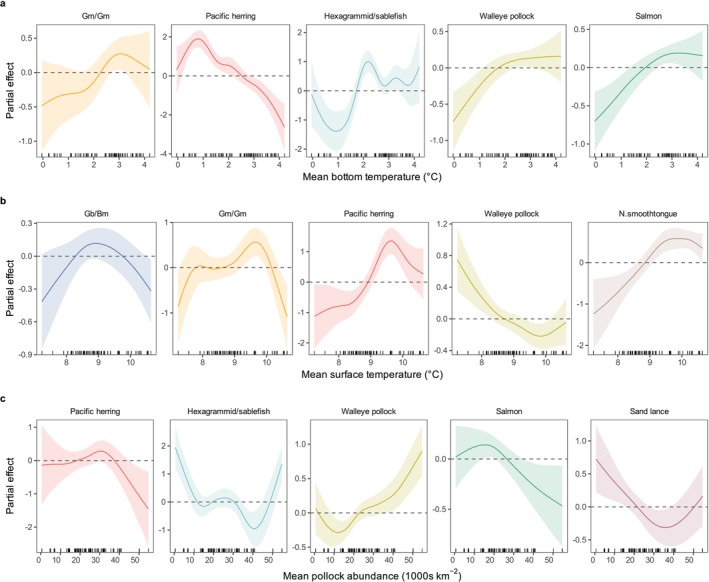
Partial effects of bottom temperature (a), surface temperature (b) and the pollock abundance index (c) on the frequency of occurrence of key prey groups in northern fur seal diets from the Pribilof Islands. The shaded area is the approximate 95% point‐wise confidence interval. Prey abbreviations: Gb/Bm = *Gonatopsis borealis/Berryteuthis magister*, Gm/Gm = *Gonatus madokai* / *G. middendorffi* , N. smoothtongue = Northern smoothtongue. Each subplot represents a different key prey group. Results are displayed on the link function scale.

All prey groups had a significant relationship with at least one of the three environmental predictor variables (Table 2, Figure 4). Pacific herring FO was highest at bottom temperatures <ca. 2°C, whereas the FO of hexagrammid/sablefish, pollock, salmon, and Gm/Gm all tended to be lower at cooler bottom temperatures (Figure 4a). Herring and northern smoothtongue were consumed more frequently at higher SSTs, whereas pollock FO generally decreased as SST increased (Figure 4b). Consumption of both squid groups was also related to SST, with a slight peak in FO around 9°C–9.5°C and decreases in FO at the lowest and highest SSTs. Pollock FO increased with the pollock abundance index, while the FO of herring, sand lance, hexagrammid/sablefish, and salmon generally decreased, at least across part of the range of pollock abundance (Figure 4c).

### 3.2 Diet and Pup Metrics

**FIGURE 5:**
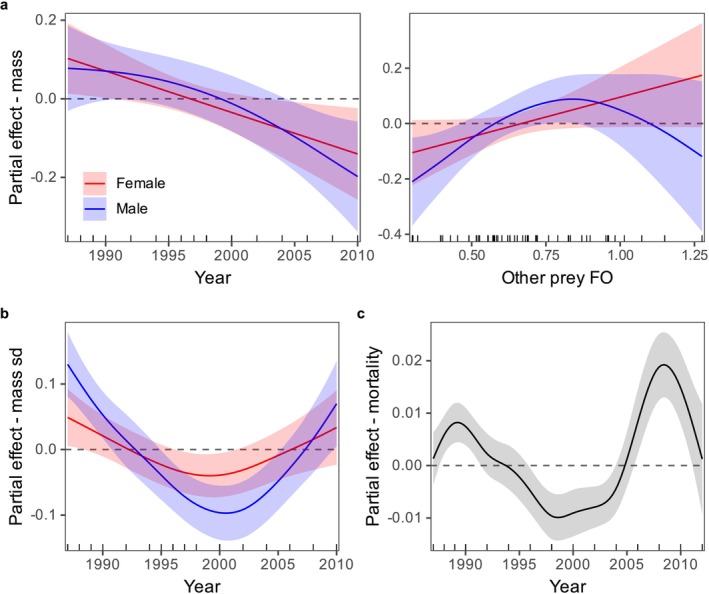
Partial effects of year and the summed FO of non‐pollock prey groups on northern fur seal August pup mass (a), pup mass standard deviation (b), and early pup mortality rates (c). The shaded area is the approximate 95% point‐wise confidence interval.

Rookery‐specific yearly mean pup masses ranged from 7.2 to 10.6 kg, with an overall average across all complexes of 8.8 kg (Table 3). The average yearly sex‐specific sample size (±SD) was 225 ± 125 pups per complex, with a range of 88–824 pups. The explained deviance of the pup mass model was 82.5%, with sex (*F* = 1103, *p* < 0.01), year, complex, and the summed FO of other prey all identified as important predictor variables for at least one sex. Most of the explained deviance was due to pup sex (95.1%) with minor contributions from the other variables. Pup mass exhibited a relatively linear decrease with year and a non‐linear increase with the FO of other prey (male pups only) (Figure 5a). For the standard deviation of pup mass model, which had an explained deviance of 60.2%, important predictor variables were sex (*F* = 324.5, *p* < 0.01, 87.7% of explained deviance) and year (9.8%), with below average variation in pup mass between ca. 1996 and 2004 (Figure 5b). The exclusion of the two SG—North data points in 1994 from the analysis did not alter the general patterns for either metric.

**TABLE 3:** Summary of total sample sizes for pup mass across all years, mean pup masses (kg), and mean pup mortality rate (%) of northern fur seals from the Pribilof Islands, separated by complex and sex (when applicable). Means represent averages of yearly rookery means, with minimum and maximum values in parentheses. Pup masses are standardized to account for variation in sample date as described in the main text.

	St. George Island		St. Paul Island		
	South	North	Reef Point	English Bay	East
Pup mass					
Number of years	5	10	11	12	15
Sample size	539 (F) 658 (M)	2025 (F) 2312 (M)	1450 (F) 1744 (M)	2349 (F) 2802 (M)	4702 (F) 5312 (M)
Female (F)	8.3 (8.0–8.8)	8.3 (7.7–9.0)	8.0 (7.3–8.8)	8.0 (7.2–8.4)	8.1 (7.2–8.9)
Male (M)	9.5 (8.9–10.0)	9.8 (9.1–10.6)	9.4 (8.5–10.2)	9.2 (8.7–9.9)	9.5 (8.8–10.3)
Mortality					
Number of years	4	12	11	11	15
Rate	2.9 (1.2–4.8)	3.1 (0.8–6.3)	4.1 (2.0–7.4)	4.8 (3.1–7.3)	3.5 (1.6–12.5)

Rookery‐specific yearly mean pup mortality rates ranged from 0.8% to 12.5%, with an overall average (±SD) of 3.7% ± 0.8% across all complexes (Table 3). Average complex‐specific means tended to follow similar patterns as the pup mass data, with higher mean mortality rates at complexes with lower mean pup masses (and vice versa). The pup mortality rate model explained 63.8% of the deviance, with complex, rookery, and year identified as important predictor variables. Rookery contributed the most to the explained deviance (48.1%) followed by year (30.7%) and complex (12.5%). The effect of year was characterized by below average mortality between ca. 1996 and 2004, followed by an increase thereafter (Figure 5c).

## 4 Discussion

We found that physical and biological conditions on the eastern Bering Sea shelf impacted the diet composition of northern fur seals from the Pribilof Islands, adding to the body of literature investigating the factors driving diet variation of marine central‐place foragers in the eastern Bering Sea (Springer et al. 2007; Sinclair et al. 2008; Renner et al. 2012; Paredes et al. 2014; Will and Kitaysky 2018). Such studies are informative for understanding how environmental changes might impact predators and trophodynamics, which is increasingly relevant given ongoing and projected physical and biological changes in the eastern Bering Sea. Year and complex still explained much of the variation for most prey groups, which likely represent “catch all” variables for relative or absolute changes in prey abundance within foraging areas as well as other factors influencing fur seal foraging behavior, such as the proximity of terrestrial rookeries to foraging habitats. While water temperatures are one of the defining features used to describe interannual variation in environmental conditions on the eastern Bering Sea shelf, they are unlikely to capture more subtle physical and biological differences that may differentiate years (Duffy‐Anderson et al. 2017; Stabeno et al. 2017). Across the years of our study, the Bering Sea shifted from a system characterized by high interannual variability in temperatures to one of stanzas with multiple sequential cold or warm years, which may further differentiate our warm and cold study years from each other, particularly as it relates to lagged environmental effects on prey population dynamics (Williams and Quinn II 2000; Heintz et al. 2013). Below, we primarily focus our discussion on potential factors driving the observed relationships between fur seal diet composition and environmental predictor variables, limiting discussion to prey groups of pollock and some forage fish species for which relationships with water temperatures are better understood.

### 4.1 Pollock Abundance Index

Walleye pollock is a key groundfish species in the eastern Bering Sea ecosystem, both as abundant prey and as a significant predator (Aydin and Mueter 2007; Buckley et al. 2016). They are arguably the most important prey for Pribilof Island northern fur seals in terms of biomass consumption (McHuron et al. 2020), and unlike some other pollock predators, fur seals consume pollock across a range of age and size classes (age‐0 to mature pollock). On the continental shelf, northern fur seals can access pollock throughout the water column, with lactating females performing epipelagic, benthic, or a mix of the two dive types on foraging trips at depths up to 191 m (Kuhn et al. 2010, 2014). Diving effort is often concentrated around the thermocline, with larger prey consumed at or below the mixed layer depth (MLD) (Kuhn 2011; Kuhn et al. 2022). Smaller prey are consumed throughout the water column; however, a greater percentage of capture attempts occur above the MLD (Kuhn et al. 2022). This behavior is consistent with ontogenetic shifts in vertical distribution of pollock, where age‐0 pollock are predominately found in the upper 50 m of the water, often around the MLD (but see Spear et al. 2023), and adult pollock typically occur below the MLD and near the bottom (Swartzman et al. 1994). Age‐1 and age‐2 pollock are vertically segregated in some years, with age‐1 occurring near the bottom and/or in dense midwater schools and age‐2 typically within the water column (Duffy‐Anderson et al. 2003). Because the pollock abundance index was derived from the bottom trawl survey data, it predominately reflects the abundance of mature and some juvenile pollock.

We found that pollock FO in fur seal diets increased with the pollock abundance index, which may reflect the effects of increased availability on consumption, either because more fur seals are likely to encounter pollock on foraging trips or because of targeted shifts in foraging locations to exploit abundant pollock. These findings are consistent with those of Joy et al. (2015) who found that the foraging behavior of females from the Reef Point complex in 2005 and 2006 was influenced by an index of pollock abundance derived from commercial fishery catches. Joy et al. (2015) suggested this relationship may have arisen because adult pollock are cannibalistic on juvenile pollock; hence, adult abundance may provide indirect information about midwater juvenile abundance. While this is a viable hypothesis, age‐1 recruitment (i.e., summer numerical abundance of age‐1 pollock for the entire stock) was exceptionally low during 2004 and 2005, which corresponded with low cannibalism rates (Boldt et al. 2012) and a much greater dependence by fur seals on mature pollock (McHuron et al. 2020). Our results thus seem to indicate that demersal pollock abundance within fur seal foraging areas directly influences the frequency of pollock consumption by fur seals, albeit with lesser importance than variables such as year. The inability to include midwater pollock abundance within the analysis does result in an incomplete understanding of how pollock availability influences fur seal prey consumption, although it is likely that it may be somewhat indirectly captured by the year effect.

The pollock abundance index was influential on fur seal consumption of several other prey groups, with declines in FO across at least part of the measured range of pollock abundance. For two of the four prey groups, these relationships appeared to primarily be driven by declines at higher pollock abundances. Declines in FO with increasing pollock abundance may reflect the dominance of pollock as a prey group on the continental shelf, with fur seals consuming other species less frequently when pollock are abundant. While the magnitude of change was small in most instances, these relationships indicate that projected declines in pollock biomass by the end of the century (Mueter et al. 2011; Holsman et al. 2020) are likely to have some impact on fur seal diet composition. Our results are also likely to underestimate the effects of reductions in pollock biomass on consumption of other prey groups because this metric does not represent midwater pollock abundance.

### 4.2 Bottom Temperature

Interannual variation in summer bottom temperatures on the eastern Bering Sea shelf is strongly influenced by the cold pool, which forms due to melting sea ice and is characterized by summer bottom water ≤ 2°C. The cold pool extends southwards along the middle shelf; during cold years, it may extend far enough south that it overlaps the foraging ranges of fur seals from St. George Island, whereas during warm years it may overlap little (or not at all) with the foraging ranges of fur seals from any complex. The occurrence of the cold pool contributes to some of the observed bottom temperature variation across complexes, as fur seals from different complexes differ in their spatial use of the continental shelf. Because the water column below 35 m is typically well mixed due to tidal activity (Stabeno et al. 2007), our bottom temperature variable is likely representative of water temperatures throughout much of the water column.

The effects of bottom temperature on pollock distribution have been relatively well described in the eastern Bering Sea (Wyllie‐Echeverria and Wooster 1998; Hollowed et al. 2012; Eisner et al. 2020; Grüss et al. 2021), which likely contributed to the observed increase in pollock FO in fur seal diets up to bottom temperatures ca. 2°C. Age‐0 and adult pollock tend to be more broadly distributed across the shelf during years with warmer bottom temperatures, which may be because their prey are more dispersed or that a reduced cold pool extent allows for more cross‐shelf movement by age classes that tend to avoid the cold pool (Hollowed et al. 2012; Eisner et al. 2020; Yasumiishi et al. 2020). Similarly, age‐1 pollock from midwater acoustic‐trawl surveys were more likely to occur across a broader range of fur seal foraging areas during warm years (Hollowed et al. 2012). Demersal age‐1 pollock captured in the bottom trawl survey tended to occur more frequently and in higher biomass across fur seal foraging ranges during years with cold bottom temperatures (Hollowed et al. 2012), but this contradictory response is likely captured by the pollock abundance index. Broader distributions of multiple pollock age classes during years with warmer bottom temperatures and reduced cold pool extents may increase the probability of more fur seals encountering pollock while foraging on the shelf. Age‐0 pollock also exhibit higher abundance and occur shallower in the water column under warmer bottom temperatures (Yasumiishi et al. 2020; Spear et al. 2023), which also could increase pollock FO due to a higher encounter probability on foraging trips or shifts in foraging locations to exploit this increased abundance. Indeed, the FO of juvenile pollock in thick‐billed murre ( *Uria lomvia* ) chick diets from St. George Island also increased with bottom temperature, which coincided with shifts in foraging locations from basin to shelf habitat based on satellite telemetry of breeding adults (Kokubun et al. 2018).

Fur seals exhibited increases in salmon FO with bottom temperature that were broadly similar to the observed pollock relationship, but uncertainty in species composition and consumption of a range of salmon age classes complicates interpretation of this relationship. Of the limited number of individuals that could be identified to species (< 4%), chum salmon ( *O. keta* ) were the most frequently occurring (70%), followed by coho ( *O. kisutch* ) and sockeye ( *O. nerka* ) salmon (10% each), pink salmon ( *O. gorbuscha* , 5%), chinook salmon ( *O. tshawytscha* , 2.5%), and Dolly Varden ( *Salvelinus malma* , 2.5%). The distribution and/or biomass of some of these species do appear to be related to warm and cold stanzas in a direction consistent with the observed relationship, which may reflect thermal preferences or responses by age‐0 pollock, one of their primary prey species during warm years (Murphy et al. 2016; Yasumiishi et al. 2020, 2024). Although perhaps less likely, it is possible that earlier migration timing from pelagic oceanic waters to freshwater rivers during warmer years could also contribute to the observed relationship with bottom temperature (Kovach et al. 2013).

Pacific herring FO exhibited a negative but non‐linear relationship with bottom temperature, with a distinct increase in consumption when bottom temperatures were < 2°C. Herring prefer relatively cool bottom temperatures (0.5°C–2.0°C) (Gunther et al. 2024) and increased herring abundance has been observed in cold years on the middle shelf, potentially because of the influence of environmental conditions on adult movement from nearshore spawning areas to overwintering grounds on the middle and outer shelf (Tojo et al. 2007; Andrews et al. 2016). Thus, the observed relationship may reflect shifts in herring spatial distribution that make them more accessible to fur seals in August during years with cold bottom temperatures while also reflecting broad scale thermal preferences of this species.

### 4.3 Surface Temperature

August sea surface temperatures (SST) within foraging areas of lactating female fur seals are influenced by winter sea ice dynamics and seasonal stratification of the water column, the magnitude of which varies temporally and spatially (Stabeno et al. 2007; Ladd and Stabeno 2012). While interannual variation in mean SSTs broadly mimicked bottom temperature patterns within fur seal foraging areas, they were not strongly correlated in our dataset and tended to be more tightly coupled with less variation among complexes than bottom temperatures.

Pacific herring biomass has been positively linked with SST (Yasumiishi et al. 2020), which is generally consistent with our observations that fur seals consumed herring more frequently above average SSTs of 9°C and lower FO below this value. Much of the Pacific herring biomass in the Bering Sea occurs in inner shelf habitat, largely outside of the foraging range of lactating females from the Pribilof Islands (Yasumiishi et al. 2020). Because of this, it is also possible that the observed relationship with SST has to do with local dynamics around the Pribilof Islands that are not necessarily reflected at broader spatial scales.

Fur seals consumed pollock less frequently as SST increased, which is not necessarily consistent with observations that age‐0 pollock biomass is positively related to SST (Yasumiishi et al. 2020). The increased biomass observed by Yasumiishi et al. (2020) may be somewhat captured by the bottom temperature variable, as mentioned previously. It is possible that the trend towards increasing pollock FO at lower surface temperatures reflects the behavior of midwater age‐1 pollock, which have the highest probability of occurrence in the vicinity of the 100 m isobath and the outer shelf and the greatest densities to the northwest of the Pribilof Islands during cold years (Hollowed et al. 2012). Lactating northern fur seals frequently occur in these areas, with a higher predicted probability of occurrence along the 100 m isobath and outer shelf during cold years (McHuron et al. 2025). While predictable concentrations of midwater age‐1 pollock are a plausible explanation for the observed relationship with SST, they are largely speculative without further separation of pollock FO into different age classes.

Northern smoothtongue is one of two key prey groups (the other being Gb/Bm) that are primarily associated with foraging on the slope and in the ocean basin (Sinclair et al. 1994). This habitat‐specific consumption was reflected in the large amount of model deviance explained by complex for these two prey groups, particularly Gb/Bm, as St. George Island is closer to the basin than St. Paul Island. Such variation has been described previously for northern fur seals and is also reflected in seabird diets from these two islands (Renner et al. 2012). Despite being associated with off‐shelf foraging, we found that smoothtongue FO was positively related to SST on the continental shelf. It seems unlikely that the observed increase in FO with SST resulted from a habitat‐induced shift from continental shelf to basin foraging by fur seals, since a similarly strong relationship was not observed for Gb/Bm. It is possible that higher SSTs may result in shifts in diel vertical migratory behavior by northern smoothtongue (Kitamura and Murata 2020) that increase their accessibility to fur seals, as fur seals in the basin almost exclusively forage nocturnally at depths < 20 m (Kuhn et al. 2014). This hypothesis is speculative, however, given the limited information on this species and because conditions on the shelf may not reflect those in the basin.

## 5 Limitations

There are well‐known biases associated with diet composition estimates from hard parts analysis, such as only reflecting the most recent meals and under‐representation of species with fragile hard parts (Bowen and Iverson 2013). Underrepresentation is likely to be less relevant for our study given the focus was on relative trends within a prey group; although it could still be influential if the within‐species bias was inconsistent across years. Using multiple methodologies may help overcome biases associated with any one method; in particular, DNA techniques would help better resolve salmon that have fragile otoliths and allow for alternative metrics of composition (e.g., biomass proportion) in a timely manner. Spatial and temporal patterns of fur seal FO data broadly reflect biomass estimates based on a more limited sample size (McHuron et al. 2020), but captive feeding studies for other pinniped species indicate it does not always accurately reflect prey consumption (Tollit et al. 2007). As a result, there is limited insight that can be gleaned about caloric consumption from FO data.

The inclusion of data from multiple rookery complexes into a single analysis increased sample sizes and the range across which environmental variables were measured. Complex‐specific smooths were not included in models because we did not feel sample sizes were sufficient to justify such complexity, which could result in over‐generalization of results. This is most likely to be a potential issue for SG—South where fur seals have a greater dependence on basin prey than other complexes. Our results also should not be generalized outside of August or lactating females, as other age classes are not as restricted in their movements, and diet compositions are similar but not necessarily equivalent (Call and Ream 2012).

As endotherms, the effects of physical environmental variation on northern fur seal diet composition are largely mediated through their influences on prey and lower trophic levels. In the absence of abundance data for most prey groups, we used surface and bottom water temperatures as proxies for prey availability; however, the importance of year in almost all analyses indicates these variables, considered alone, are not entirely sufficient to explain interannual variation in diet composition. A multivariate index of physical environmental variables that also incorporates lagged environmental variables might be more appropriate, particularly given local (and regional) variables tend to be somewhat correlated. We also treated prey abundance and temperature as separate variables, but environmental variables can have various direct and indirect mechanisms for influencing diet composition. For example, surface temperature could have a direct influence via prey vertical availability (temperature → FO), but also an indirect effect via impacts on prey spatial distribution (temperature → prey abundance → FO). Extending our analysis by using GAMs within path analysis (e.g., using “piecewiseSEM” Lefcheck 2016) could estimate the total effect for a covariate given these direct and indirect mechanisms, which has relevance for climate forecasts of prey abundance and their resulting impacts on predator foraging success.

## 6 Implications of Diet Variation

Interannual variation in pup metrics was largely unexplained by diet composition. There was a slight increase in male pup weights as the summed FO of other prey in the diet increased, at least across the lower range of the data. The general trend for female pups was similar, although it was not considered to be an important variable under the study criteria. This increase suggests that the availability of other prey groups is somewhat beneficial to lactating female fur seals from the Pribilof Islands. Availability of non‐pollock prey could be beneficial for a variety of reasons, such as reducing intra‐specific competition, buffering seals from fluctuations in pollock availability, or increasing the caloric density of female diets. The comparatively small influence of diet composition does not necessarily mean that prey does not influence pup growth and survival. It is possible that limitations associated with FO (e.g., it does not reflect numerical abundance) or the data used in this study hindered the ability to detect relationships. For example, there was considerable variability in early mortality rates associated with individual rookeries, even within a complex, but FO data were calculated at the complex level due to sample size limitations. In addition, we were unable to account for other factors that are known to influence early mortality and pup growth rates, such as maternal foraging trip durations (Calambokidis and Gentry 1985; Trites 1992; Merrill et al. 2021). There were some commonalities in temporal trends across several pup metrics that largely coincided with the temporal trends observed in pollock FO and some other prey groups. These commonalities could indicate that the underlying drivers are related to pollock in some way, although further investigation is needed to better elucidate the factors contributing to interannual and spatial trends in fur seal pup mass and early mortality rates.

## 7 Conclusions

Our results indicate that projected environmental changes in the eastern Bering Sea are likely to influence fur seal diet composition, with the potential for small effects on fur seal pup mass. Pollock was the primary prey group consumed at almost all complexes across the study years, highlighting the importance of this species to Pribilof Island fur seals. Not only was pollock consumed more frequently as it became more abundant within fur seal foraging areas, but its relative abundance also affected how frequently other prey groups were consumed. Pollock biomass is projected to decline considerably by 2100 and shift northwards (Holsman et al. 2020; Rooper et al. 2021), which may place more trophic pressure by fur seals on other groundfish, forage fish, salmon, or squid populations in the eastern Bering Sea. It is difficult, however, to use our results in any sort of predictive capacity without the ability to resolve the underlying drivers of the observed temporal and spatial effects on diet composition. Although temporal trends are likely ultimately driven by regional and local physical environmental conditions that translate through the food web, water temperatures alone had limited ability to resolve much of the variation in fur seal diet composition. While our dataset included cold and warm years, it is more than a decade out of date, and the eastern Bering Sea has experienced several marine heatwaves and a period of record‐low sea ice since 2012. Analyzing diet data collected post‐2012 and incorporating newer diet methodologies would allow for a more contemporary and complete measure of diet composition and facilitate the consideration of additional biological predictor variables, such as midwater juvenile pollock abundance. It would also provide insight into how diet composition changed during a time when water temperatures were largely at the upper extent or outside of the range of what was included in our analysis.

## Author Contributions

**Elizabeth A. McHuron:** conceptualization (equal), formal analysis (lead), writing – original draft (lead), writing – review and editing (lead). **Jeremy T. Sterling:** data curation (equal), investigation (equal), writing – review and editing (supporting). **Katie Luxa:** data curation (equal), investigation (equal), writing – review and editing (supporting). **James Thorson:** formal analysis (supporting), writing – review and editing (supporting). **Rod Towell:** data curation (equal), investigation (equal), writing – review and editing (supporting). **Rolf R. Ream:** investigation (equal), writing – review and editing (supporting). **Tonya Zeppelin:** conceptualization (equal), investigation (equal), writing – review and editing (supporting).

## Conflicts of Interest

The authors declare no conflicts of interest.

## Supporting information


**Data S1:** ece371998‐sup‐0001‐DataS1.docx.

## Data Availability

The data and code that support the findings of this study are openly available on Github (https://github.com/emchuron/nfs‐diet‐environment) and Zenodo (McHuron 2025).
