# Supplementary material for: Biological and Physical Environmental Drivers of Diet Variation in Northern Fur Seals
Source: Ecol Evol. 2025 Aug 18;15(8):e71998. doi: 10.1002/ece3.71998 (PMC12361816; doi:10.1002/ece3.71998)
Supplement: Supplementary file 1 — Data S1: ece371998‐sup‐0001‐DataS1.docx. [file ECE3-15-e71998-s001.docx]

**Biological and physical environmental drivers of diet variation in northern fur seals**

**Appendix: Supporting figures and tables**

Figure A1. Terrestrial locations of the five northern fur seal rookery complexes on St. George and St. Paul Islands.

Figure A2. Example of the effects of sample size on frequency of occurrence for two key prey groups, walleye pollock (a) and salmon (b) in a single year. Individual diet samples were randomly selected (with replacement) to create sample sizes that ranged from 1 - 100 for each complex and year combination. Frequency of occurrence was calculated for each sample size. This process was replicated 100 times. Data are represented as boxplots with upper and lower whiskers calculated as described in the function description for *geom_boxplot*. The red dot in each subplot represents the actual sample size and FO for the year and the dashed vertical red line the sample size cutoff used in the analysis.

Table A1. Prey taxon detected in fur seal scat, spew, and enema samples and prey group classification.

| **Taxon name** | **Common name** | **Prey group** |
| --- | --- | --- |
| Actinopterygii |  | Fish |
| Agonidae |  | Poacher |
| Bathyagonus sp. |  | Poacher |
| Blepsias bilobus | Crested sculpin | Poacher |
| Blepsias sp. |  | Poacher |
| Ammodytes spp. | Sand lance | Sandlance |
| Anarhichadidae |  | Wolffish |
| Anarhichas orientalis | Bering wolffish | Wolffish |
| Anoplopoma fimbria | Sablefish | HexSable |
| Leuroglossus schmidti | Northern smoothtongue | Smoothtongue |
| Lipolagus ochotensis | Eared blacksmelt | Smoothtongue |
| Clupea pallasii | Pacific herring | Herring |
| Sardinops sagax | Pacific sardine | Herring |
| Cottidae |  | Sculpin |
| Gymnocanthus sp. |  | Sculpin |
| Hemilepidotus sp. | Irish lord sp. | Sculpin |
| Aptocyclus ventricosus | Smooth lumpsucker | Lumpsucker |
| Gadus chalcogrammus | Walleye pollock | Pollock |
| Gadus macrocephalus | Pacific cod | Cod |
| Gadus sp. |  | Gadus |
| Gasterosteus aculeatus | Threespine stickleback | Stickleback |
| Hexagrammidae |  | HexSable |
| Hexagrammos sp. |  | HexSable |
| Pleurogrammus monopterygius | Atka mackerel | HexSable |
| Hexagrammidae/Anoplopomatidae |  | HexSable |
| Pleurogrammus monopterygius/Anoplopoma fimbria | Atka mackerel/Sablefish sp. | HexSable |
| Careproctus sp. |  | Snailfish |
| Liparidae |  | Snailfish |
| Liparis sp. |  | Snailfish |
| Merluccius productus | Pacific hake | Hake |
| Diaphus theta | California headlightfish | Myctophid |
| Lampanyctus jordani | Brokenline lanternfish | Myctophid |
| Myctophidae |  | Myctophid |
| Nannobrachium regale | Pinpoint lampfish | Myctophid |
| Protomyctophum thompsoni | Northern flashlightfish | Myctophid |
| Stenobrachius leucopsarus | Northern lampfish | Myctophid |
| Stenobrachius nannochir | Garnet lampfish | Myctophid |
| Stenobrachius sp. |  | Myctophid |
| Tarletonbeania crenularis | Blue lanternfish | Myctophid |
| Mallotus villosus | Capelin | Smelt |
| Osmeridae |  | Smelt |
| Thaleichthys pacificus | Eulachon | Smelt |
| Atheresthes sp. | Arrowtooth/Kamchatka flounder sp. | Arrowtooth |
| Hippoglossoides elassodon | Flathead sole | Flatfish |
| Hippoglossus stenolepis | Pacific halibut | Flatfish |
| Lepidopsetta sp. | Rock sole sp. | RockSole |
| Pleuronectiformes |  | Flatfish |
| Oncorhynchus gorbuscha | Pink salmon | Salmon |
| Oncorhynchus keta | Chum salmon | Salmon |
| Oncorhynchus kisutch | Coho salmon | Salmon |
| Oncorhynchus nerka | Sockeye salmon | Salmon |
| Oncorhynchus tshawytscha | Chinook salmon | Salmon |
| Salmonidae |  | Salmon |
| Salvelinus malma | Dolly Varden | Salmon |
| Sebastes sp. |  | Rockfish |
| Leptoclinus maculatus | Daubed shanny | Prickleback |
| Stichaeidae |  | Prickleback |
| Trichodon trichodon | Pacific sandfish | Sandfish |
| Zaprora silenus | Prowfish | Prowfish |
| Zoarcidae |  | Eelpout |
| Cephalopoda |  | Cephalopod |
| Decapodiformes |  | Squid |
| Gonatidae |  | Gonatid |
| Gonatopsis borealis/Berryteuthis magister |  | Gb/Bm |
| Gonatus berryi |  | Gonatid |
| Gonatus madokai/middendorffi |  | Gm/Gm |
| Gonatus onyx |  | Gonatus |
| Gonatus pyros |  | Gonatus |
| Gonatus sp. |  | Gonatid |
| Gonatus tinro |  | Gonatus |
| Octopoda |  | Octopus |
| Chondrichthyes |  | Chondrich |
| Rajidae |  | Skate |
| Squalus acanthias | Spiny dogfish | Shark |
| Lampetra tridentata | Pacific lamprey | Lamprey |
| Petromyzontidae |  | Lamprey |
| Polychaeta |  | Polychaeta |
